# The prognostic landscape of genes and infiltrating immune cells in cytokine induced killer cell treated-lung squamous cell carcinoma and adenocarcinoma

**DOI:** 10.20892/j.issn.2095-3941.2021.0023

**Published:** 2021-08-30

**Authors:** Jian Wang, Fan Yang, Qian Sun, Ziqing Zeng, Min Liu, Wenwen Yu, Peng Zhang, Jinpu Yu, Lili Yang, Xinwei Zhang, Xiubao Ren, Feng Wei

**Affiliations:** 1 Department of Immunology, Tianjin Medical University Cancer Institute and Hospital, National Clinical Research Center for Cancer, Key Laboratory of Cancer Prevention and Therapy, Tianjin, Tianjin’s Clinical Research Center for Cancer, Key Laboratory of Cancer Immunology and Biotherapy, Tianjin 300060, China; 2 Department of Biotherapy, Tianjin Medical University Cancer Institute and Hospital, National Clinical Research Center for Cancer, Key Laboratory of Cancer Prevention and Therapy, Tianjin, Tianjin’s Clinical Research Center for Cancer, Key Laboratory of Cancer Immunology and Biotherapy, Tianjin 300060, China; 3 Cancer Molecular Diagnostics Core, Tianjin Medical University Cancer Institute and Hospital, National Clinical Research Center for Cancer, Key Laboratory of Cancer Prevention and Therapy, Tianjin, Tianjin’s Clinical Research Center for Cancer, Key Laboratory of Cancer Immunology and Biotherapy, Tianjin 300060, China

**Keywords:** NSCLC, CIK treatment, DFS, HLA class II, infiltrating immune cells

## Abstract

**Objective:**

Patients with non–small cell lung cancer (NSCLC) respond differently to cytokine-induced killer cell (CIK) treatment. Therefore, potential prognostic markers to identify patients who would benefit from CIK treatment must be elucidated. The current research aimed at identifying predictive prognostic markers for efficient CIK treatment of patients with NSCLC.

**Methods:**

Patients histologically diagnosed with NSCLC were enrolled from the Tianjin Medical University Cancer Institute and Hospital. We performed whole-exome sequencing (WES) on the tumor tissues and paired adjacent benign tissues collected from 50 patients with NSCLC, and RNA-seq on tumor tissues of 17 patients with NSCLC before CIK immunotherapy treatment. Multivariate Cox proportional hazard regression analysis was used to analyze the association between clinical parameters and prognostic relevance. WES and RNA-seq data between lung squamous cell carcinoma (SCC) and adenocarcinoma (Aden) were analyzed and compared.

**Results:**

The pathology subtype of lung cancer was the most significantly relevant clinical parameter associated with DFS, as analyzed by multivariate Cox proportional hazard regression (*P* = 0.031). The patients with lung SCC showed better CIK treatment efficacy and extended DFS after CIK treatment. Relatively low expression of HLA class II genes and checkpoint molecules, and less immunosuppressive immune cell infiltration were identified in the patients with lung SCC.

**Conclusions:**

Coordinated suppression of the expression of HLA class II genes and checkpoint molecules, as well as less immune suppressive cell infiltration together contributed to the better CIK treatment efficacy in lung SCC than lung Aden.

## Introduction

Lung cancer, the leading cause of cancer-related mortality worldwide, is associated with more than 1.5 million deaths every year^1^. Most patients present with locally advanced or metastatic disease. Approximately 85% of lung cancers are classified as non–small cell lung cancer (NSCLC), including lung adenocarcinoma (Aden), squamous cell carcinoma (SCC), and large cell carcinoma histologic subtypes. Over the past decade, major advances in the understanding of lung cancer, particularly NSCLC, have been achieved^2^. Molecular targeted cancer therapy has achieved great progress in improving the overall survival (OS) rate in NSCLC. Epidermal growth factor receptor (EGFR) has been identified as an oncogenic driver. Blockade of EGFR with specific tyrosine kinase inhibitors (TKIs) generates dramatic tumor responses and has shown significant efficacy in the treatment of patients carrying specific mutations in the EGFR TK domain in their tumor cells^1,3–5^. These molecular alterations are influential in Aden and have important implications in lung cancer treatment^6^. Currently, none of the recurrent molecular alterations commonly present in lung SCC have been demonstrated to be as predictive of the response to therapy as EGFR alterations in lung Aden. Despite the establishment of tumor profiling for lung adenocarcinomas, its clinical benefits for other histologic subtypes of lung cancer, such as lung SCC, remain unclear. Furthermore, a minority of patients diagnosed with NSCLC have EGFR mutations and typically develop resistance within 9 to 12 months^7^. The more complex genomic mutation features in patients with SCC often result in a failure to benefit from EGFR mutation based molecular targeted lung cancer therapy.

Cancer immunotherapy is an emerging treatment taking advantage of improving the anti-cancer ability of immune cells by reconstituting the host immunity usually suppressed by tumor cells^8^. Among the different types of immunotherapy, cytokine-induced killer cell (CIK) immunotherapy eliminates cancer cells through transfusion of *in vitro* expanded and activated immune cells. Several recent clinical trials have shown that CIK immunotherapy significantly improves the OS and disease-free survival (DFS) rates in patients with NSCLC^9,10^. The combination of CIK immunotherapy and chemotherapy is particularly efficient in treating multiple cancers^11–13^. However, as with many immunotherapy approaches, the clinical benefits of CIK immunotherapy for NSCLC varies among patients. Predicting which patients with specific pathology subtypes and immune profiles will benefit from CIK immunotherapy remains a great challenge needing elucidation.

Discovery of markers predictive of clinical benefits is essential for the rational design of treatment strategies in cancer immunotherapy. Furthermore, predictive markers can aid in understanding the mechanisms through which host immunity combats cancer cells. Genomic and transcriptomic features of tumors, tumor associated cells, and their microenvironment are promising candidates for predictive and prognostic biomarkers. PD-L1 expression has been shown to be a predictive biomarker for checkpoint blockade cancer immunotherapy^14^. However, several clinical studies have suggested that the tumor mutation burden (TMB) is a better predictive marker than PD-L1 expression for cancer immunotherapy^15,16^. In addition to molecular features, infiltration of tumors by immune cells has been found to be relevant to immunotherapy efficacy^17^.

In the current study, to understand how different subtypes of patients with NSCLC respond to CIK immunotherapy and to identify the markers predictive of clinical benefit in patients with NSCLC, we performed whole-exome sequencing (WES) and transcriptomic analyses of tumor tissues and paired adjacent benign tissues collected from patients before CIK immunotherapy. Integrative analysis of the WES and RNA-seq data revealed that suppression of the intestinal immune network for immunoglobulin A (IgA) production pathway, decreased expression of checkpoint molecules, and diminished immuno-suppressive cell infiltration are the hallmarks of lung SCC compared with Aden. Further analysis of the intestinal immune network for IgA production pathway revealed that suppressed expression of human leukocyte antigen (HLA) class II genes was associated with better efficacy of CIK treatment in patients with lung SCC. We further developed our approach by defining a set of multiple immune cell subpopulations and checkpoint molecules, thus providing a comprehensive view of the cellular composition of intra-tumoral immune infiltration. Our current study presents an alternative solution for patients with suppressed immune response, facilitates the rational design of immunotherapy, and provides a broad scope for understanding the molecular features mediating the response of NSCLC to immunotherapy.

## Materials and methods

### Patient selection

This study was approved by the National Medical Products Administration (China) (2006L01023) and by the Ethics Committee of the Tianjin Medical University Cancer Institute and Hospital (Approval No. E2016055), according to the guidelines of the Declaration of Helsinki. All patients provided signed informed consent before entering the study. Patients who were eligible for enrollment were required to meet the following criteria: age between 18 and 75 years, pathologically confirmed NSCLC (according to the 7th edition of the Cancer Staging Manual of the American Joint Committee on Cancer)^18^, an Eastern Cooperative Oncology Group (ECOG) performance status score of 0 or 1^19^, no measurable lesion after operation according to version 1.1 of the Response Evaluation Criteria in Solid Tumors (RECIST)^20^, and an expected survival duration of ≥ 3 months. Patients were excluded if they had immune deficiency or autoimmune diseases, other malignancies, severe allergic disorders, or uncontrollable medical conditions, or if they were pregnant or lactating. According to the patient selection criteria, a total of 50 patients histologically diagnosed with NSCLC were enrolled from Tianjin Medical University Cancer Institute and Hospital. Detailed information on the patients can be found in **Supplementary Table S1**.

### CIK preparation

Autologous CIKs were prepared as described in our previous studies^21,22^. Briefly, peripheral blood mononuclear cells were collected from patients with NSCLC with a Code Spectra Apheresis System (Caridian BCT, Lakewood, USA). The cells were cultured in medium containing 50 ng/mL anti-CD3 antibody (e-Bioscience, San Diego, USA), 100 U/mL recombinant human interleukin (IL)-1α, and 1,000 U/mL interferon-γ (IFN-γ) to induce the CIK cells, at 37 °C under 5% CO_2_ for 24 h. Subsequently, 300 U/mL of recombinant human IL-2 was added to the medium, and the medium was regularly replaced with fresh IFN-γ- and IL-2-containing medium every 5 days. All products were free of contamination with bacteria, fungi, and mycoplasma, and contained < 5 endotoxin units. On day 14, the CIK cells were harvested, and the median number of CIK cells was 7.4 × 10^9^, with a viability greater than 95%. This method led to enrichment of the CD3^+^CD56^+^ cellular subset.

### Study design and treatment

Patients received chemotherapy in combination with autologous CIK cell immunotherapy after operation. Patients received TP regimen (paclitaxel, 135 mg/m^2^, day 1; cisplatin, 80 mg/m^2^, day 1), GP regimen (gemcitabine, 1,000 mg/m^2^, days 1 and 8; cisplatin, 80 mg/m^2^, day 1), or NP regimen (navelbine, 25 mg/m^2^, days 1 and 8; cisplatin, 80 mg/m^2^, day 1), and autologous CIK infusion (day 15, 16, total count of CIK cells ≥ 1 × 10^10^) of each cycle, with 4 weeks per cycle for 4 cycles. CIK treatment continued at an interval of 1 month until disease progression occurred.

### High throughput sequencing

A total of 0.6 µg genomic DNA per sample was used as input material for the DNA sample preparation. Sequencing libraries were generated with an Agilent SureSelect Human All Exon V6 kit (Agilent Technologies, Santa Clara, CA, USA) according to the manufacturer’s recommendations, and index codes were added to each sample. The clustering of the index-coded samples was performed on a cBot Cluster Generation System by using a Hiseq PE Cluster Kit (Illumina) according to the manufacturer’s instructions. After cluster generation, the DNA libraries were sequenced on the Illumina Hiseq platform, and 150 bp paired-end reads were generated. A total of 2 µg RNA per sample was used as input material for the RNA sample preparation. Sequencing libraries were generated with an NEBNext^®^ UltraTM RNA Library Prep Kit for Illumina^®^ (NEB, Ipswich, MA, USA) according to the manufacturer’s recommendations, and index codes were added so that sequences could be associated with each sample. The clustering of the index-coded samples was performed on a cBot Cluster Generation System by using a TruSeq PE Cluster Kit v3-cBot-HS (Illumina) according to the manufacturer’s instructions. After cluster generation, the library preparations were sequenced on the Illumina Hiseq platform, and 125 bp/150 bp paired-end reads were generated.

### Sequencing data analysis

For WGS data, valid sequencing data were mapped to the reference human genome (UCSC hg19) with Burrows-Wheeler Aligner (BWA) software^23^ to obtain the original mapping results stored in BAM format. If one read in a pair mapped to multiple positions, the strategy adopted by BWA was to choose the most likely placement. If 2 or more most likely placements were present, BWA chose one randomly. Then SAMtools^24^ and Picard (http://broadinstitute.github.io/picard/) were used to sort BAM files and perform duplicate marking, local realignment, and base quality recalibration to generate the final BAM files. GATK (v3.4) software^25^ was used to perform SNP calling.

For RNA sequencing data, clean paired-end reads were aligned to the human reference genome (hg19) with Hisat2 (v2.0.5)^26^, as guided by the gene model Ensembl GRCh37.87. HTSeq (v0.11.2) was used to count the read numbers mapped to each gene.

### Differential expression analysis and pathway enrichment

Differential expression analysis was performed with the DESeq2 package (v1.18.1)^27^ in R version 3. The resulting *P*-values were adjusted with Benjamini and Hochberg’s approach for controlling the false discovery rate. Genes with an adjusted *P*-value < 0.05 found by DESeq2 were assigned as differentially expressed. Kyoto Encyclopedia of Genes and Genomes (KEGG) pathway enrichment analysis of differentially expressed genes was implemented in the cluster Profiler R package (v3.6.0)^28^. KEGG terms with a corrected *P*-value less than 0.1 were considered significantly enriched in differentially expressed genes.

### Analysis of immune cell infiltration

Gene set enrichment analysis (GSEA) was performed to identify cell types corresponding to the 2 CD4^+^ T-cell subclusters of the 10x Genomics PBMC dataset. We performed GSEA with the Bioconductor R package fgsea (v1.4.0) and analysis of gene sets for 64 immune and stroma cell types with the R package xCell (v1.1.0). For each DE method, the input to fgsea was a list of genes ranked by a test statistic comparing expression in the 2 CD4^+^ T-cell subclusters^29,30^.

### Statistical analysis

The Cox proportional hazard regression model was fitted with the coxph function with the R package survival (v2.41-3), by considering cancer subtype, TMB, age, and sex as the covariates.

## Results

### Differential response of lung SCC and Aden to CIK-based immunotherapy

**Figure 1:**
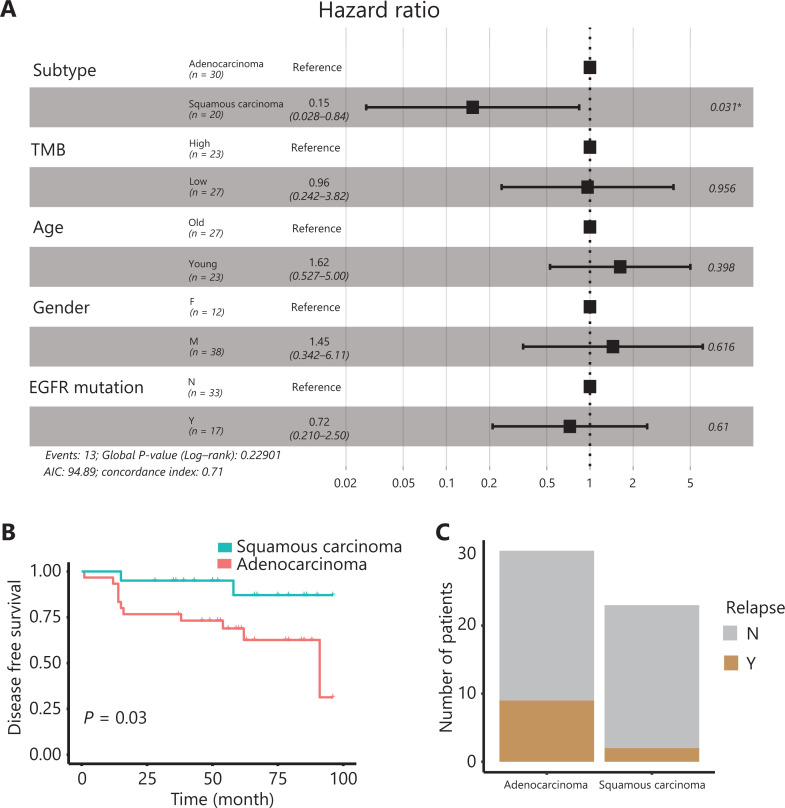
Differential response of lung SCC and Aden to CIK immunotherapy. A. Multivariate Cox proportional hazard regression analysis of clinical parameters. B. Kaplan-Meier survival analysis of the disease-free survival (DFS) rates in lung SCC (*n* = 20) and Aden (*n* = 30) after CIK therapy. C. The 5-year relapse events of lung SCC and Aden after CIK therapy. *P < 0.05.

**Figure 2:**
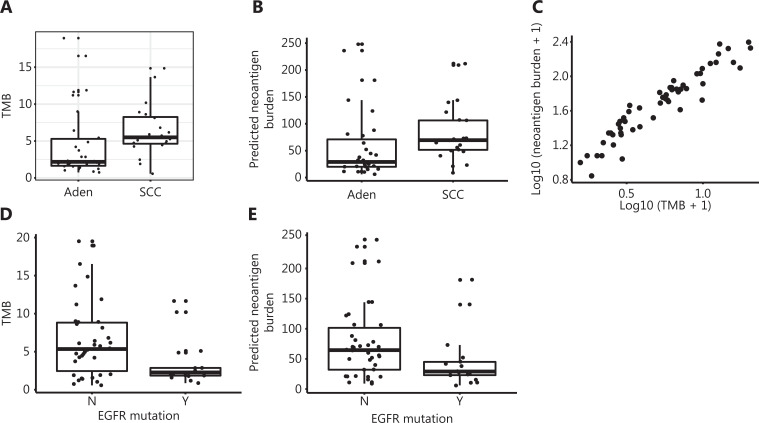
Differential TMB and neoantigen burden in lung SCC and Aden. A. Tumor mutation burden (TMB) of lung SCC and Aden, calculated from whole-exome sequencing data of tumor tissues. B. Predicted neoantigen burden in lung SCC and Aden. C. Neoantigen burden correlates with TMB in lung cancer. D. Patients with EGFR mutation have lower TMB. E. Patients with EGFR mutation have lower neoantigen burden.

We performed WES on tumor tissues and paired adjacent benign tissues collected from 50 patients with NSCLC and performed RNA-seq on tumor tissues from 17 patients with different types of lung cancer before CIK immunotherapy treatment (**Supplementary Table S1**). To explore the association between the described clinical parameters and CIK treatment efficacy, we used multivariate Cox proportional hazard regression analysis to analyze the prognostic relevance. The NSCLC subtype emerged as a significant prognostic variable in the multivariate analysis (**Figure 1A**). Molecular targeted cancer therapy is less effective in lung SCC^31^. Unexpectedly, patients with lung SCC treated with CIK immunotherapy showed longer DFS than those with lung Aden (Kaplan-Meier survival curves, log rank test, *P* = 0.03) (**Figure 1B**). The 5-year relapse rate was also lower in patients with lung SCC (8.7%) than those with lung Aden (29.0%) (two-tailed Fisher’s exact test, *P* = 0.092) (**Figure 1C**). Although TMB is a predictive marker for checkpoint inhibitor cancer immunotherapy^16^, it was not found to be a significant prognostic variable (**Figure 1A**). However, the TMB calculated from somatic nonsynonymous mutations was higher in patients with lung SCC than lung Aden (*P* = 0.01348) (**Figure 2A**), thus suggesting a hidden link between TMB and the efficacy of CIK treatment.

### TMB correlates with computationally predicted neoantigens

Several lines of evidence suggest that neoantigens targeted by T cells mediate clinical responses to cancer immunotherapy^32,33^. In agreement with previous reports^34^, higher nonsynonymous mutations in lung SCC were associated with high computationally predicted neoantigens, as revealed in our exome sequencing data (*t*-test, *P* = 0.071) (**Figure 2B**). The association of TMB with neoantigen burden was not restricted to lung cancer subtype, and we found that TMB highly correlated with the computationally predicted neoantigen burden in the whole cohort (R-square 0.90, *P* < 2e-16) (**Figure 2C**). EGFR mutations are present at high frequency in patients with lung cancer who are non-smokers^35^. The EGFR mutation rate was lower in patients with SCC (two-tailed Fisher’s exact test, *P* = 0.017) (**Supplementary Figure S1**). We found that patients with lower EGFR mutation rates had a higher TMB (*t*-test, *P* = 0.0031) and higher predicted neoantigen burden (*t*-test, *P* = 0.0201) (**Figure 2D–2E**). Although the complex genomic mutation landscape in SCC hinders molecular targeted therapy, large numbers of neoantigens may facilitate the recognition of tumor cells by CIKs.

### HLA class II gene expression is a prognostic marker of CIK treatment response in NSCLC

**Figure 3:**
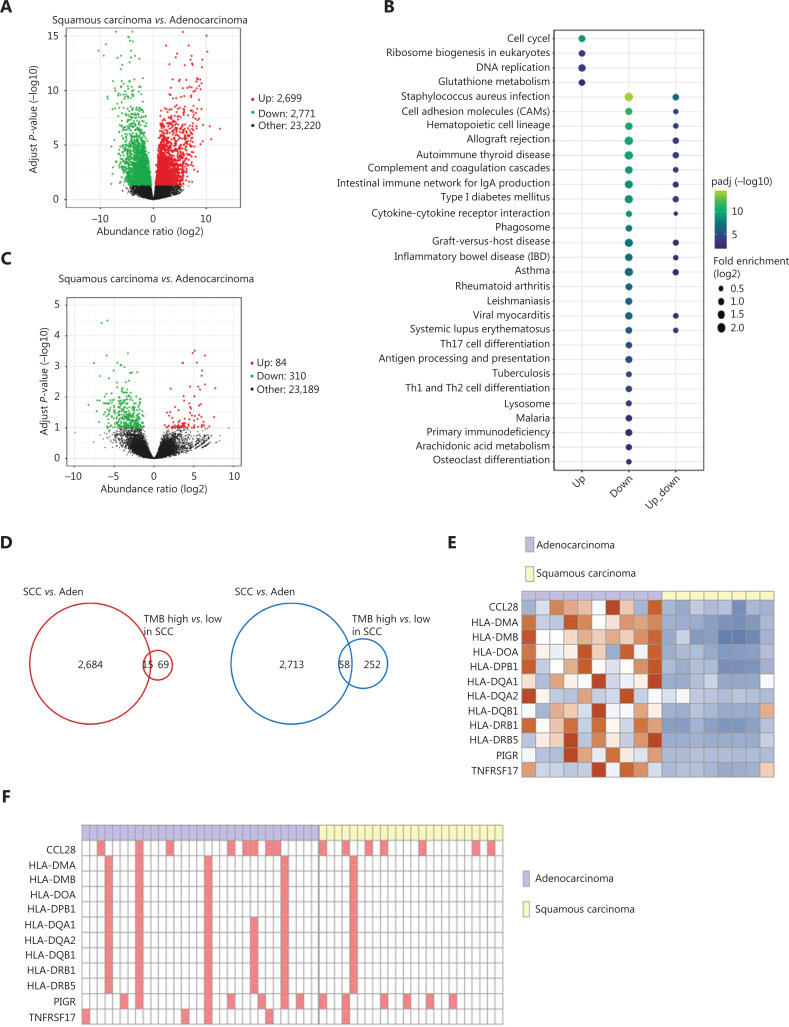
Transcriptomic analysis of lung SCC and Aden tumor tissues. A. Volcano plot of transcriptomic differences between lung SCC and Aden; The red and green dots represent genes up-regulated and down-regulated in SCC compared with Aden. The cut-off for differentially expressed genes was an adjusted *P*-value < 0.05. B. KEGG pathway enrichment analysis of differentially expressed genes between SCC and Aden. Up indicates pathways enriched in the up-regulated genes in SCC, whereas down indicates the down-regulated ones; Up-down indicates pathways enriched in differentially expressed genes regardless of regulation direction. C. Volcano plot of transcriptomic differences between patients with lung SCC with high and low TMB. D. Venn diagram showing overlapping differentially expressed genes in the SCC *vs*. Aden and high *vs*. low TMB comparisons. E. Expression levels of MHC class II genes in lung SCC and Aden. F. Copy number gain events of MHC class II genes in lung SCC and Aden.

To further examine the molecular mechanisms underlying the better CIK treatment efficacy in lung SCC, we compared the transcriptomes of tumor tissues collected from 17 patients with NSCLC. A total of 2,699 genes were up-regulated, whereas 2,771 genes were down-regulated in patients with lung SCC compared with lung Aden at the cutoff *P*-value < 0.05 (**Figure 3A**). More than 5,000 differentially expressed genes between SCC and Aden tumor tissues revealed distinct gene expression features. Genes highly expressed in SCC were enriched in the functions of DNA replication, cell cycle and protein translation pathways, thus implying more active tumor cell proliferation in patients with SCC. In contrast, genes down-regulated in SCC were significantly enriched in functions of immune related pathways, such as the intestinal immune network for IgA production, cytokine-cytokine receptor interaction, and complement and coagulation cascades (**Figure 3B**). To explore whether TMB might be associated with transcriptomic changes between patients with SCC and Aden, we further compared the transcriptomes of patients with high and low TMB in the SCC group. In the patients with high TMB, more genes (310 genes) were down-regulated than up-regulated (84 genes) (**Figure 3C**). KEGG pathway enrichment analysis showed similar enriched terms for genes that were less abundant in patients with high TMB, such as the intestinal immune network for IgA production and T helper cell differentiation pathways (**Supplementary Figure S2**). Furthermore, genes that were down-regulated in the high TMB group were enriched in other immune related pathways, including the antigen processing and presentation pathway, B cell receptor signaling pathway, and natural killer cell mediated cytotoxicity (**Supplementary Figure S2**). These results collectively suggested that patients with lung SCC exhibited impaired immune functions or diminished immune cell infiltration, which was more severe in patients with high TMB. Furthermore, the differentially expressed genes in patients with lung SCC *vs.* Aden significantly overlapped with those between the high and low TMB groups (*P* = 1.1e-3 for up-regulated genes; *P* = 1.5e-10 for down-regulated genes) (**Figure 3D**), thus implying a link between TMB and the differences in transcriptomic and immune function between lung SCC and Aden.

The main function of the intestinal immune network for IgA production is to generate noninflammatory IgA antibodies, which serve as the first line of defense against microorganisms and disease, and are an indicator of immune function. The HLA class II genes in the intestinal immune network for IgA production pathway were expressed at low levels in patients with lung SCC (**Supplementary Figure S3, Figure 3E**). Analysis of copy number variation in our cohorts revealed more copies of HLA class II genes in lung Aden (**Figure 3F**). The HLA class II members HLA-DRA, HLA-DQA1, HLA-DQB1, and HLA-DOB were also weakly expressed in the high TMB group of patients with SCC (**Figure 3D**), thus suggesting that higher TMB in patients with SCC may result in less recruitment of immune cells expressing HLA class II genes. Another key component in this pathway, B-cell maturation antigen (BCMA), which is preferentially expressed in mature B lymphocytes, was also weakly expressed in patients with lung SCC (**Supplementary Figure S3**), thus suggesting widely suppressed immune responses in tumor tissue from patients with SCC.

**Figure 4:**
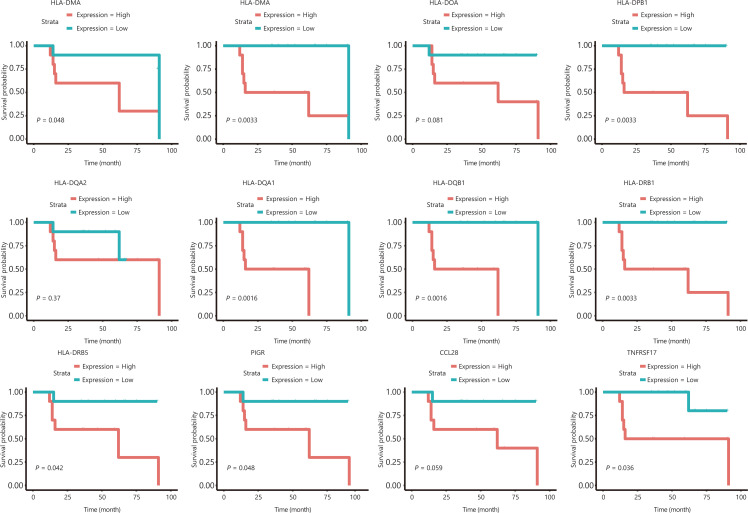
Gene expression levels in the intestinal immune network for IgA production pathway are predictive markers of DFS after CIK therapy.

We further explored whether suppressed expression of HLA class II and other genes in the intestinal immune network for IgA production pathway in patients with SCC might be associated with better prognosis for CIK treatment. We then divided all patients into 2 groups according to the expression level of these genes without taking cancer type into consideration, then compared the DFS in these 2 groups. Low expression of HLA class II genes (DMA, DMB, DOA, DPB1, DQA2, DQA1, DQB1, DRB1, and DRB5) was associated with prolonged DFS. Similarly, low expression of B-cell maturation antigen (BCMA/TNFRSF17), chemokine ligand 28 (CCL28), and the polymeric immunoglobulin receptor (PIGR) was also associated with better efficacy of CIK treatment (**Figure 4**). The association between DFS and immune related gene patterns was further supported by the comparison of the transcriptomes in patients with long and short DFS (**Supplementary Figure S4A–S4B**). We compared the transcriptomes of patients who did not relapse until after 5 years and those who relapsed within 3 years after CIK immunotherapy. We observed suppression of immune response pathways in patients who did not relapse until after 5 years (**Supplementary Figure S4A–S4B**).

### Correlation between infiltrating immune cells and HLA class II gene expression

**Figure 5:**
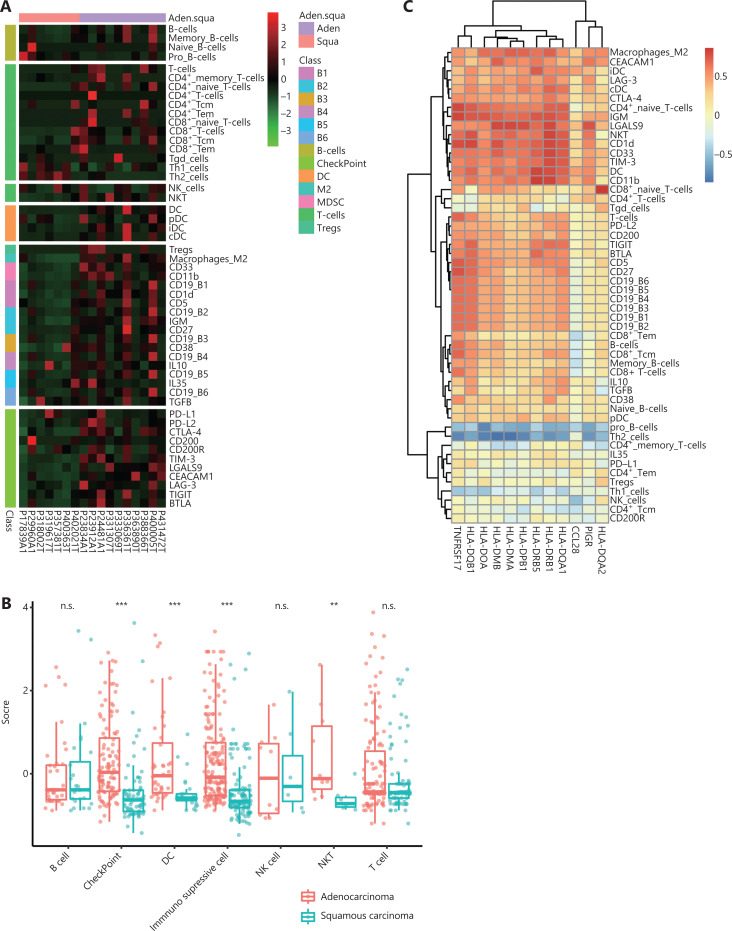
Tumor immune cell infiltration in lung SCC and Aden. A. Heat map of the immune cell abundance in patients, inferred from the expression levels of marker genes. B. The statistical amounts of different types of tumor immune cell infiltration and expression levels of checkpoint molecules in lung SCC and Aden. C. Correlation of MHC class II genes with immune cell infiltration and checkpoint molecule expression. **P < 0.01, and ***P < 0.001.

Tumor infiltrating immune cells play important roles in determining the tumor microenvironment and prognosis. Inspired by the diminished expression of immune pathway genes in patients with SCC and their prognostic significance for CIK treatment, we analyzed the abundance of different subsets of immune cells and checkpoint molecules in tumor tissues from patients with lung SCC or Aden. We estimated multiple subpopulations of immune cells, including B cells (memory B cells, naïve B cells, and pro-B cells), T cells (CD4^+^ T cells, CD4^+^ naïve T cells, CD4^+^ Tcm, CD4^+^ Tem, CD8^+^ T, cells, CD8^+^ naïve T cells, CD8^+^ Tcm, CD8^+^ Tem, Th1, Th2, and Tγδ), NK cells, NKT cells, DCs, and immune suppressive cells (such as Tregs, TAM, MDSCs, and Bregs). The checkpoint molecules PD-L1, PD-L2, CTLA-4, CD200, CD200R, TIM-3, LGALS9, CEACAM1, LAG-3, TIGIT, and BTLA were also detected in tumor tissues. The tumor infiltrating immune cells were highly heterogeneous among patients, whereas some common features were shared within subtypes (**Figure 5A**). The amount of tumor infiltrating B cells was similar in patients with lung SCC *vs.* Aden, and the infiltrating T cells differed slightly, with no statistical significance (**Figure 5B**). Strikingly, the immune suppressive cells and checkpoint molecules were dramatically lower in patients with SCC than those with Aden (**Figure 5A and 5B**); this effect might be responsible for the better CIK treatment efficacy of patients with lung SCC. In addition, the expression level of HLA class II genes was indeed strongly associated with the checkpoint molecules CEACAM1, LAG-3, CTLA-4, TIM-3, and LGALS9. Furthermore, the numbers of DCs and NKT cells resident in tumor tissues was highly correlated with the expression levels of HLA class II genes. All these results collectively demonstrated that the entire tumor immune suppressive microenvironment might be responsible for the better efficacy of CIK treatment. This possibility was also further supported by the immune scores for lung SCC and Aden (**Supplementary Figure S5**).

### The tumor immune microenvironment contributes to differences in CIK efficacy in patients with NSCLC

We then attempted to determine the main mechanisms through which patients with SCC benefited from CIK immunotherapy. We analyzed the expression levels of chemokines such as CCLs and CXCLs, and found that almost all types of chemokines were up-regulated in the Aden patient samples (**Supplementary Figure S6A and S6B**). CCL17 and CCL22 within tumors are known to be associated with an increased population of Foxp3^+^ Tregs; thus, the high expression of these 2 chemokines may contribute to the infiltration of Tregs into patients with Aden, thereby making the tumor microenvironment more immune suppressive. In addition, CXCL16 and CXCL17 have been reported to be positively correlated with M2-macrophage infiltration, enhanced angiogenesis, and poor prognosis in cancer. Thus, high expression of CXCL16 and CXCL17 may also contribute to the formation of an immune suppressive tumor microenvironment in patients with Aden. Overall, high expression of chemokines in patients with Aden may easily attract more immune suppressive cells such as Tregs and M2 cells, thus leading to poorer CIK efficacy in patients with Aden. Moreover, we observed high expression of major histocompatibility complex (MHC) I as well as MHC II in patients with Aden, which may lead to the inhibitory cytotoxicity functions of the T-NK phenotype (CD3^+^CD56^+^) of most CIKs (**Supplementary Figure S6C and S6D**). Members of the UL16 binding proteins (ULBP) family and MHC class I chain-related molecules (MIC) A/B are known to be natural killer group 2 member D (NKG2D) ligands. NKG2D is an activating receptor expressed primarily on cells in the cytotoxic arm of the immune system. During NK cell development, engagement of NKG2D has long-term effects on the expression of NK cell receptors and their responsiveness to extracellular cues, thus suggesting a role in NK cell education. Our analysis showed almost no difference in MICA/B between SCC and Aden. However, ULBP1–3 was more highly expressed in SCC than Aden, thus leading to the high cytotoxicity of CIKs (**Supplementary Figure S7A and S7B**).

### Recurrent mutations in lung cancer and their association with CIK efficacy

The mutational landscape of lung cancer has been reported^36^. A similar set of genes with high frequency mutation was also discovered from the current whole exome sequencing data (**Supplementary Figure S8A and S8C**). The mutational frequencies of different genes in the current study were comparable to those in previous studies (**Supplementary Figure S8B and S8D**). We next asked whether recurrent gene mutations might be associated with DFS in CIK treatment. Among the high frequency mutated genes, only CCT8L2 mutation was significantly associated with DFS in CIK treatment (*P* = 0.01); however, this association was supported by only 5 mutation cases. USH2A (*P* = 0.13) and LRP1B (*P* = 0.14) gene mutations were weakly associated with DFS in CIK treatment (**Supplementary Figure S9**).

## Discussion

### Differential efficacy of CIK immunotherapy in lung SCC *vs.* Aden

Molecular targeted therapies against receptor tyrosine kinases have shown strong responses in subsets of patients with lung Aden with genomic alterations in several kinase genes, including EGFR, ALK, and ROS1. However, the mutational profile of lung SCC is often more complicated, and the rate of smoking is high in these patients. The higher TMB and neoantigen burden hinder the development of drugs for molecular targeted therapy but simultaneously provide an opportunity for immunotherapy. Blockade of the immune checkpoint molecules CTLA-4 plus PD-1 has been shown to extend DFS in patients with NSCLC, depending on the TMB^15^. TMB has been reported to be a novel biomarker with promising value in predicting the response to immune checkpoint inhibitors in NSCLC^37^. However, the TMB distribution in EGFR mutant patients with NSCLC is not well demonstrated, and the effects of TMB on the outcomes of cell therapies such as CIK immunotherapy have not been explored. In our study, we found that patients with lung SCC with lower EGFR mutation rates, higher TMB (*t*-test, *P* = 0.0031), and higher predicted neoantigen burden (*t*-test, *P* = 0.0201) (**Figure 2D and 2E**) benefitted from CIK immunotherapy. Some studies have shown that TMB is negatively associated with clinical outcomes in patients with metastatic EGFR-mutant lung cancer treated with EGFR TKI, including OS^38,39^. The mechanism underlying why patients with low EGFR mutation have high TMB is not well understood. One hypothesis is that higher TMB may represent a greater diversity of preexisting subclones, some of which emerge under the selective pressure of TKI therapy. An innate propensity toward mutagenesis in cancers with higher TMB might result in greater heterogeneity at the time of resistance. A growing number of articles emphasize that immunotherapy that stimulates the immune system and enhances immunological surveillance function may be a promising alternative way to treat cancers^40,41^. Adoptive cell-based immunotherapy, in this context, uses procedures stimulating immune effector cells to better recognize and finally eliminate cancer cells. In such an immunotherapeutic approach, CIKs are currently emerging as a promising and effective treatment option, particularly when combined with standard therapy in an adjuvant treatment setting^42^. The first report in the literature and the first phase I trial, performed by Schmidt-Wolf et al.,^43^ have corroborated the high cytotoxic activity of this new type of antitumor effector cell and highlighted their favorable safety and tolerability profile. CIKs can be generated easily from peripheral blood lymphocytes through sequential *ex vivo* incubation with monoclonal antibodies against CD3, as well as IFN-γ, and IL-2 in a time-sensitive schedule. The time-controlled administration of IFN-γ before the addition of IL-2 and anti-CD3 creates a high cytotoxic potential^43^. The complementary addition of IL-2 and anti-CD3 afterward principally promotes mitogenic stimulants^44^. Among the heterogeneous CIK population, the CD3^+^CD56^+^ subset is primarily responsible for the antitumor efficacy. These terminally differentiated CD3 and CD56 double-positive CIK cells develop from former CD56-negative T cells and exhibit non-MHC restricted cytolytic activity against several tumor targets^45,46^. Several clinical trials and systemic reviews have shown that combined chemotherapy with CIKs, as first-line or second-line therapies, may be effective in improving survival and quality of life in patients with advanced lung cancer^47–49^. To date, the prognostic and predictive markers for CIK immunotherapy on the 2 main subtypes of NSCLC have not been fully evaluated. In this study, we showed that CIK immunotherapy led to better efficacy in patients with lung SCC. We then further explored the potential biomarkers in CIK-treated patients with lung SCC.

### Differential immune microenvironments in lung SCC and Aden

Investigation of gene expression among patients who differentially respond to immunotherapy should facilitate the discovery of prognostic markers. Our transcriptomic analysis of patients with lung SCC or Aden revealed that the intestinal immune network for IgA production pathway was less enriched in patients with lung SCC. Previously published articles have shown that high-avidity IgA protects against bacterial enteropathogens by directly neutralizing virulence factors or through poorly defined mechanisms that physically impede bacterial interactions with the gut tissues (‘immune exclusion’)^50–53^. In previous reports, the intestinal immune network for IgA production signaling pathway has been demonstrated to be involved in the proliferation and migration of hepatocellular carcinoma cells^54^. We then investigated alterations in essential genes in the intestinal immune network for IgA production pathway. The HLA system is a set of genes encoding MHC proteins, which are responsible for regulating the immune system. MHC class I presents peptide from inside the cell, whereas MHC class II presents antigens from outside the cell to CD4^+^ T-lymphocytes^55^. CIKs are a heterogeneous immune cell population containing a high percentage of cells with a mixed T-NK phenotype (CD3^+^CD56^+^). Cytotoxicity mediated by CD3^+^CD56^+^ T cells depends on the expression of MHC molecules on tumor cells and the activation of signaling pathways through the NKG2D cell-surface receptor. NKG2D ligands are members of the ULBP family and MICA/B, which are highly expressed on tumor cells. The target cell recognition is based on the interaction between NKG2D with its ligands. Thus, the lysis of tumor cells is induced by perforin and granzyme granules released by CIKs^56,57^. Our analysis of ULBP family and MICA/B molecules described above showed that ULBP1–3 were relatively higher in patients with SSC than in patients with Aden. The highly expressed NKG2D ligands might interact with NKG2D expressed on the CD3^+^CD56^+^ T cell subpopulation of CIKs and thus enhance CIK cytotoxicity. In addition, our transcriptomic analysis demonstrated that patients with lung SCC expressed relatively lower MHC class II genes than patients with Aden. Thus, we speculated that some other mechanism independent of the NKG2D signaling pathway might exist.

We compared the immune cell infiltration and checkpoint molecule expression in tumor tissues from patients with lung SCC or Aden. The immune suppressive cells and checkpoint molecules were dramatically lower in patients with SCC than in patients with Aden. The less suppressive tumor microenvironment might have been responsible for the better efficacy of CIK treatment in patients with lung SCC. Subsequently, we found that HLA II gene expression was strongly associated with the checkpoint molecules CEACAM1, LAG-3, and CTLA-4. Of note, the TIM-3/LGALS9 pathways, which are associated with immunosuppression and poorer clinical outcomes, are also associated with lower expression of MHC class II genes. Our previous studies have shown that CIKs are characterized by high expression of TIM-3. Thus, the low expression of LGALS9 in tumor tissues from patients with SCC might weakly interact with TIM-3 expressed on CIKs and finally facilitate CIK treatment efficacy in patients with SCC. Furthermore, the amounts of DCs and NKT cells resident in tumor tissues are highly correlated with the expression level of HLA class II genes. Together, the tumor microenvironment, less infiltration of immune suppressive cells, and diminished checkpoint molecule expression allowed for the maintenance of anti-tumor activity of CIKs.

Checkpoint blockade-based immunotherapies are among the most commonly used strategies in cancer therapy, and many clinical trials have been conducted. MHC genes in tumor cells are essential for successful checkpoint blockade-based immunotherapy, because of the requirement for MHC presentation in the approach^58^. However, cancer cells are often equipped with immune evasion mechanisms by impairing MHC expression^59^. Our data emphasized the advantage of CIK immunotherapy when the expression of MHC protein is aberrant. Immune checkpoint inhibitors are efficient only when the checkpoint molecules are highly expressed. We showed that low MHC expression is associated with low checkpoint gene expression, thus suggesting that CIK treatment may serve as an important alternative approach.

### Genomic landscape of lung SCC and Aden

The 2 NSCLC subtypes of lung SCC and Aden have both unique and shared genomic, clinical, and histopathological characteristics. Using WES analysis, we identified recurrent mutations in these 2 pathological subtypes. Significantly mutated genes including TP53, TTN, and USH2A were found in patients with lung SCC. In contrast, in patients with lung Aden, in addition to TP53 and USH2A, we observed a high frequency of mutations in KRAS and EGFR. The mutational landscape observed in our study is consistent with those described in previous reports^36,60^. Although some mutations were associated with prognosis in NSCLC, very few were associated with CIK immunotherapy efficacy in the current study. In our cohort, the TMB and resultant neoantigen burden were significantly higher in lung SCC than in Aden, probably because of the high proportion of smokers among the patients with SCC^61^. The weak relationship between CIK efficacy and specific somatic mutations does not diminish the importance of the general mutational landscape and immune microenvironment in CIK treatment.

## Conclusions

In the current study, we showed that CIK immunotherapy leads to better efficacy in patients with lung SCC than in patients with lung Aden. We further explored the potential biomarkers for CIK treatment efficacy in patients with lung SCC. Coordinated suppression of the expression of HLA class II genes and checkpoint molecules, and diminished immune suppressive cell infiltration simultaneously contributed to better efficacy of CIK treatment in lung SCC than in lung Aden.
