# Supplementary material for: The prognostic landscape of genes and infiltrating immune cells in cytokine induced killer cell treated-lung squamous cell carcinoma and adenocarcinoma
Source: Cancer Biol Med. 2021 Aug 30;18(4):1134–47. doi: 10.20892/j.issn.2095-3941.2021.0023 (PMC8610154; doi:10.20892/j.issn.2095-3941.2021.0023)
Supplement: Supplementary file 1 [file cbm-18-1134-s001.pdf]

Supplementary materials

Table S1 Information for 50 patients with NSCLC

ID	Gender	Age	Type	Stage	State	Relapse	DFS	TMB	RNA-seq	EGFR mutation
P15530A1	M	54	SCC	IIA	Alive	N	117	8.133673688	N	N
P17839A1	M	59	SCC	IIIA	Alive	N	90	6.804021132	Y	N
P18713A1	M	76	SCC	IIB	Alive	N	109	4.258352839	N	N
P19909A1	M	68	Aden	IIB	Dead	Y	105	1.858102371	N	Y
P20088A1	M	53	SCC	IIIA	Alive	N	105	4.745140239	N	N
P23834A1	F	48	Aden	IIA	Dead	Y	15	2.025802319	Y	N
P23912A1	F	72	Aden	IB	Alive	Y	91	1.959586578	Y	Y
P24481A1	M	45	Aden	IIA	Dead	Y	14	11.1983717	Y	N
P24839A1	F	57	Aden	IA	Alive	N	88	1.564853024	N	N
P24907A1	M	56	Aden	IIB	Alive	N	88	18.94111867	N	N
P25310A1	M	50	Aden	IIIA	Dead	Y	14	2.085361648	N	Y
P25683A1	M	48	Aden	IA	Alive	N	85	0.742921518	N	N
P25974A1	M	67	SCC	IIA	Alive	N	86	8.620332994	N	N
P26438A1	M	56	Aden	IIB	Alive	N	84	1.79639746	N	Y
P26640A1	M	55	SCC	IB	Alive	N	85	14.85073565	N	N
P28072A1	M	61	Aden	IIA	Alive	N	79	1.911993568	N	N
P28074A1	M	75	SCC	IA	Alive	N	79	8.907607181	N	N
P28132A1	F	67	Aden	IA	Alive	N	78	2.864549189	N	Y
P29960A1	M	54	SCC	IA	Alive	N	75	5.093054453	Y	Y
P318002T	M	59	SCC	IIB	Alive	N	67	4.952073139	Y	N
P319617T	M	64	SCC	IIB	Alive	N	66	4.455692149	Y	N
P320477T	F	54	Aden	IA	Alive	N	66	1.176218857	N	Y
P326808T	M	56	SCC	IIIA	Alive	Y	58	6.168972366	N	N
P326977T	M	69	Aden	IA	Alive	N	63	1.589822276	N	Y
P331307T	M	46	Aden	IIIA	Dead	Y	62	2.842310272	Y	Y
P332036T	M	68	Aden	IIB	Alive	N	61	5.432208541	N	N
P333069T	F	58	Aden	IB	Alive	N	60	1.875568391	Y	Y
P333253T	M	63	Aden	IA	Alive	N	60	11.65148552	N	Y
P336361T	F	59	Aden	IA	Missing	N	59	0.863568365	Y	Y
P344685T	M	44	Aden	IA	Alive	N	56	16.52550675	N	N
P349588T	M	64	Aden	IIIA	Dead	Y	54	4.208936458	N	N
P355826T	M	58	Aden	IIIA	Alive	N	52	11.88076455	N	N
P357381T	M	58	SCC	IA	Alive	N	52	5.264112381	Y	N

Table S1 Continued

ID	Gender	Age	Type	Stage	State	Relapse	DFS	TMB	RNA-seq	EGFR mutation
P361258T	F	53	SCC	IIIA	Alive	N	50	1.897254152	N	N
P363890T	F	52	Aden	IA	Alive	Y	12	2.312744938	Y	Y
P367497T	F	50	Aden	IIIA	Alive	N	49	3.728924293	N	N
P374031T	M	64	Aden	IIB	Alive	N	46	1.411787679	N	N
P378361T	M	65	SCC	IIA	Dead	Y	15	4.691127319	N	N
P382931T	M	59	Aden	IA	Alive	N	53	2.270946191	N	Y
P382977T	M	56	SCC	IA	Alive	N	43	13.66078992	N	N
P388366T	M	65	Aden	IIIB	Alive	Y	14	1.025072011	Y	N
P392196T	F	59	SCC	IA	Alive	N	39	2.47026344	N	Y
P395628T	M	61	Aden	IB	Dead	Y	1	6.419426543	N	N
P397285T	M	64	Aden	IB	Alive	Y	38	8.998396387	N	N
P400005T	F	62	Aden	IV	Alive	N	37	1.223366103	Y	N
P400363T	M	56	SCC	IB	Alive	N	35	10.18914784	Y	Y
P402021T	M	38	SCC	IA	Alive	N	36	0.571884843	Y	N
P430491T	M	72	SCC	IB	Alive	N	28	5.897974118	N	N
P431472T	M	54	Aden	IA	Alive	Y	16	4.863215214	Y	Y
P9121_1067_1	M	57	SCC	IA	Alive	Y	119	5.725887144	N	N

NSCLC, non–small cell lung cancer; Aden, adenocarcinoma; SCC, squamous cell carcinoma; LCC, large cell carcinoma; OS, overall survival; EGFR, epidermal growth factor receptor; TKIs, tyrosine kinase inhibitors; CIKs, cytokine-induced killer cells; DFS, disease-free survival; TMB, tumor mutation burden; BCMA, B-cell maturation antigen; CCL28, chemokine ligand 28; RTKs, receptor tyrosine kinases; IFN-γ, interferon-γ; NKG2D, natural killer group 2 member D.

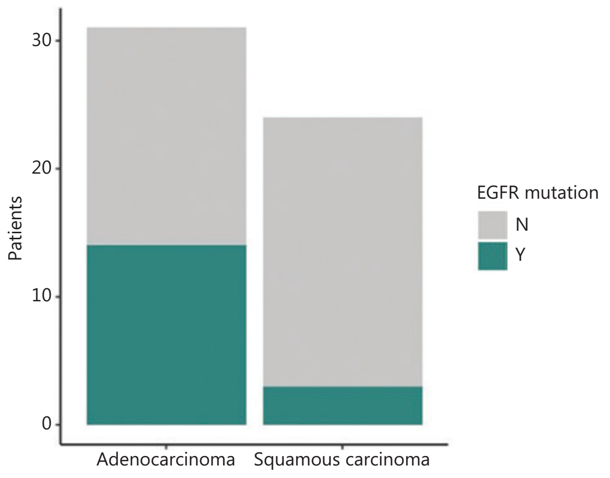

Figure S1 Number of patients with or without EGFR mutation in the cohort.

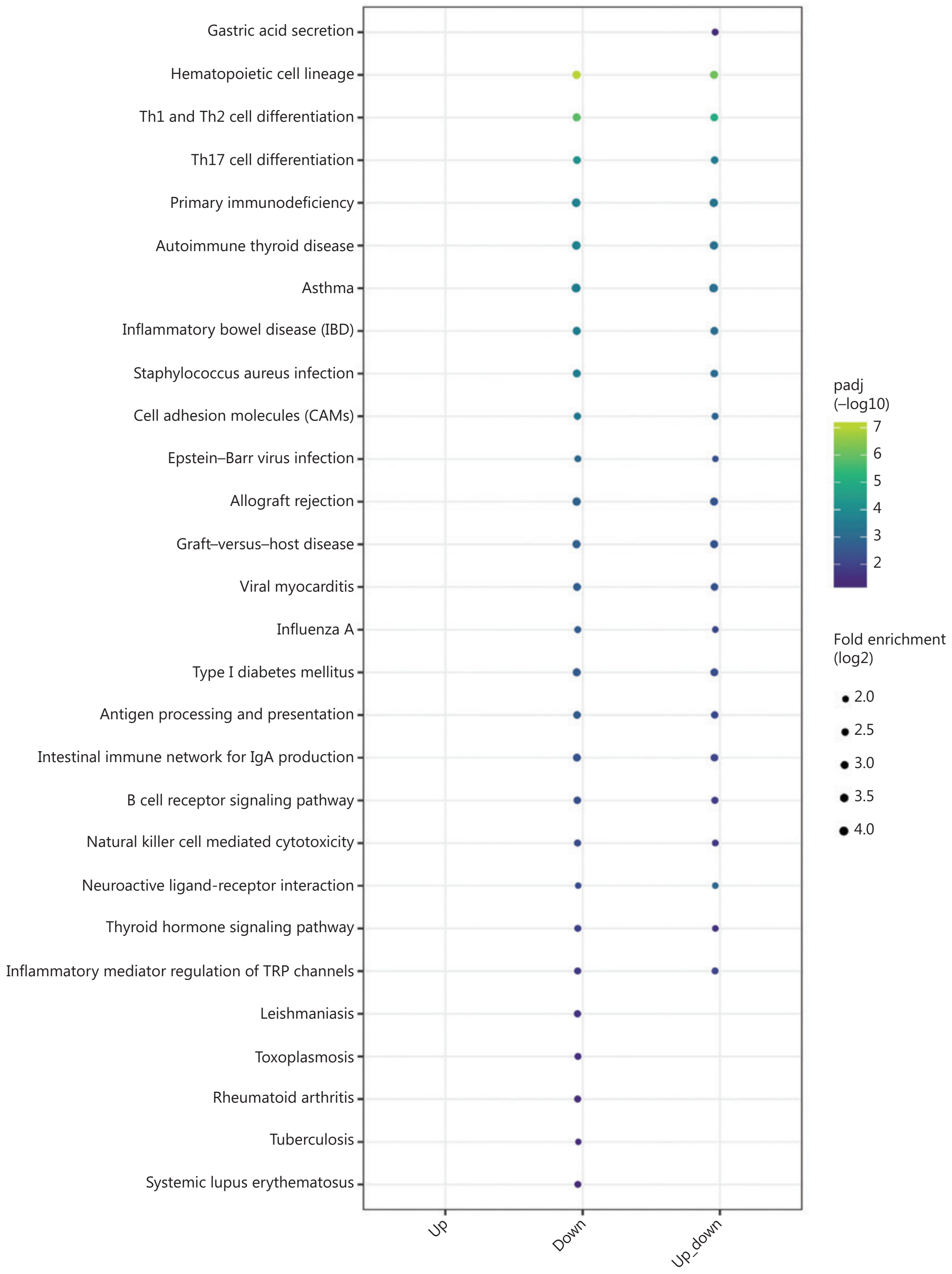

Figure S2 KEGG pathway enrichment analysis of differentially expressed genes between high and low TMB groups in patients with SCC.

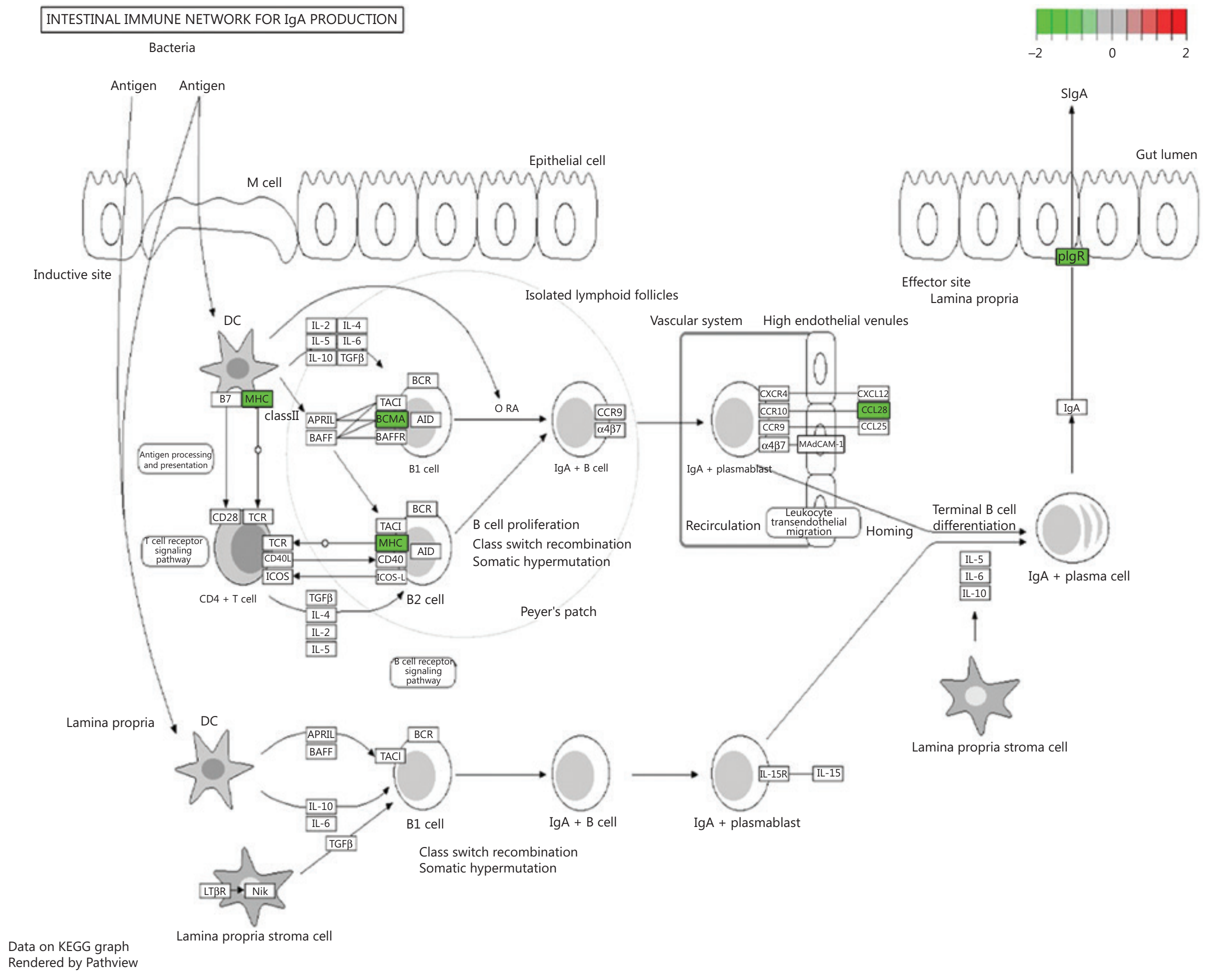

Figure S3 Schematic of the intestinal immune network for IgA production pathway. Genes differentially expressed between lung SCC and Aden are in green.

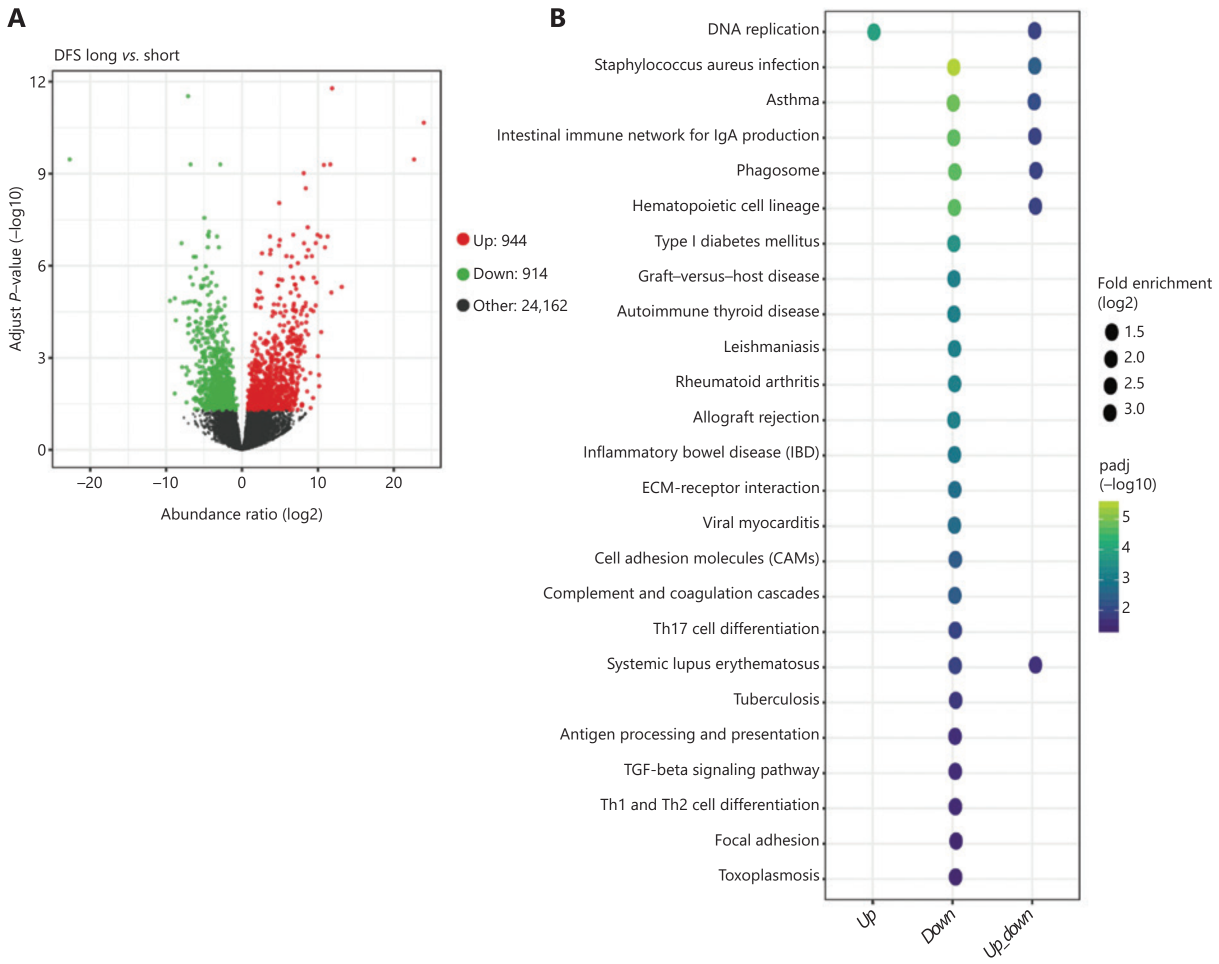

Figure S4 KEGG pathway enrichment analysis of the differentially expressed genes in patients with long and short DFS. A. Volcano plot of differentially gene expression in tumor tissues from patients with long and short DFS after therapy. B. KEGG pathway enrichment analysis of the differentially expressed genes.

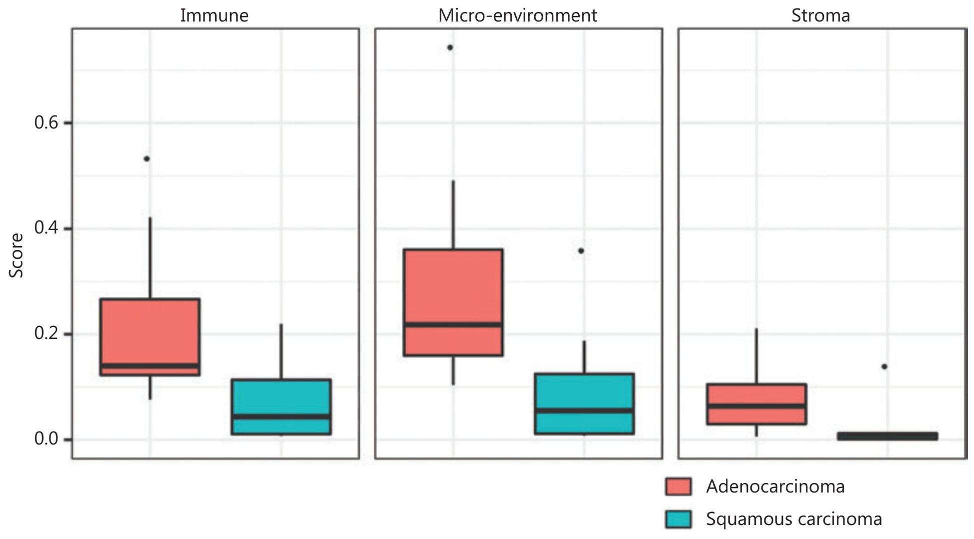

Figure S5 Immune scores in lung SCC and Aden.

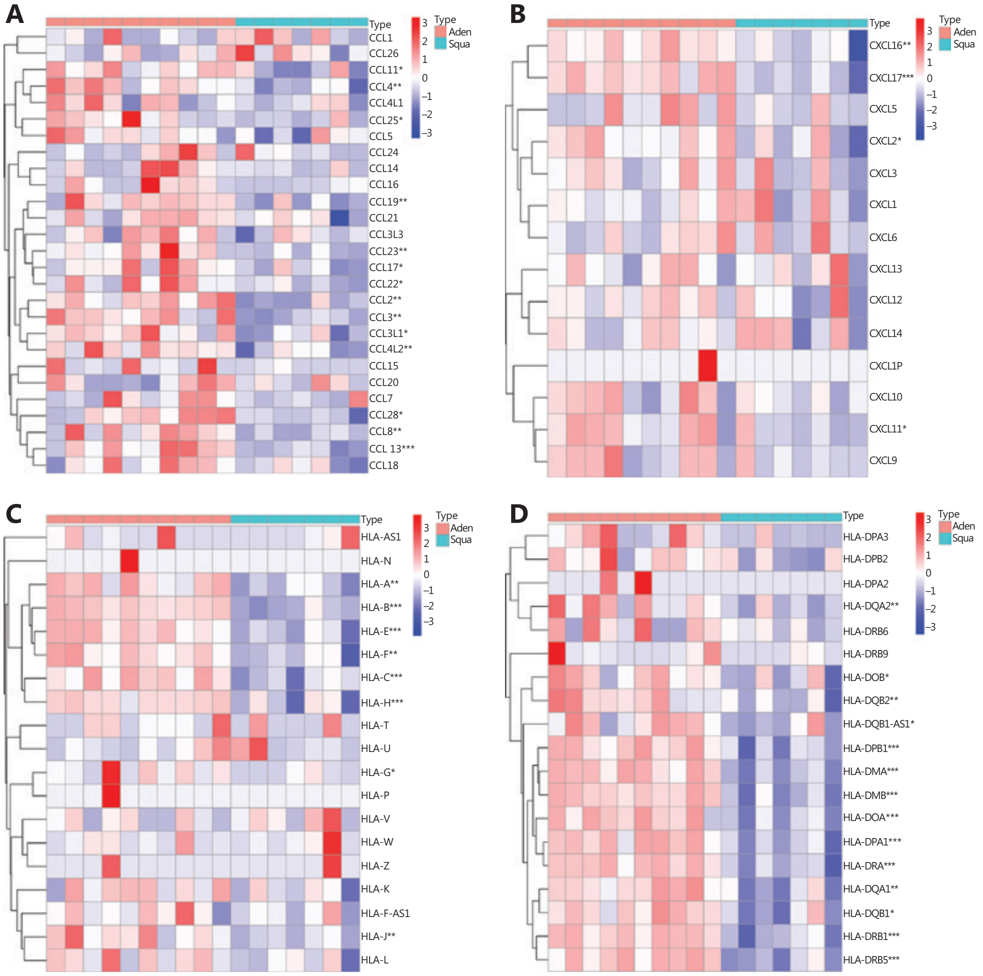

Figure S6 Expression levels of chemokines and MHC molecules in lung SCC and Aden. A. Heatmap of expression of CCLs in SCC and Aden. B. Heatmap of expression of CXCLs in SCC and Aden. C. Heatmap of expression of MHC I in SCC and Aden. D. Heatmap of expression of MHC II in SCC and Aden. *$P < 0.05$, **$P < 0.01$, and ***$P < 0.001$.

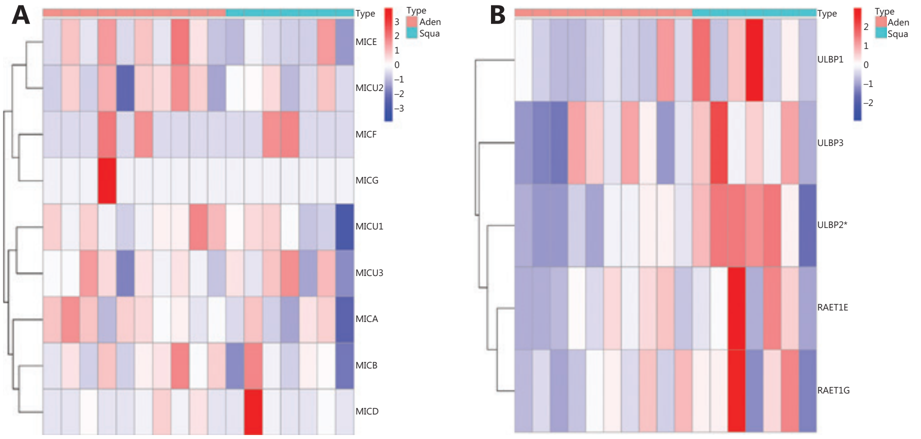

Figure S7 Expression levels of NKG2D ligands in lung SCC and Aden. A. Heatmap of expression of MICA/B in SCC and Aden. B. Heatmap of expression of ULBP family proteins in SCC Aden. *$P < 0.05$.

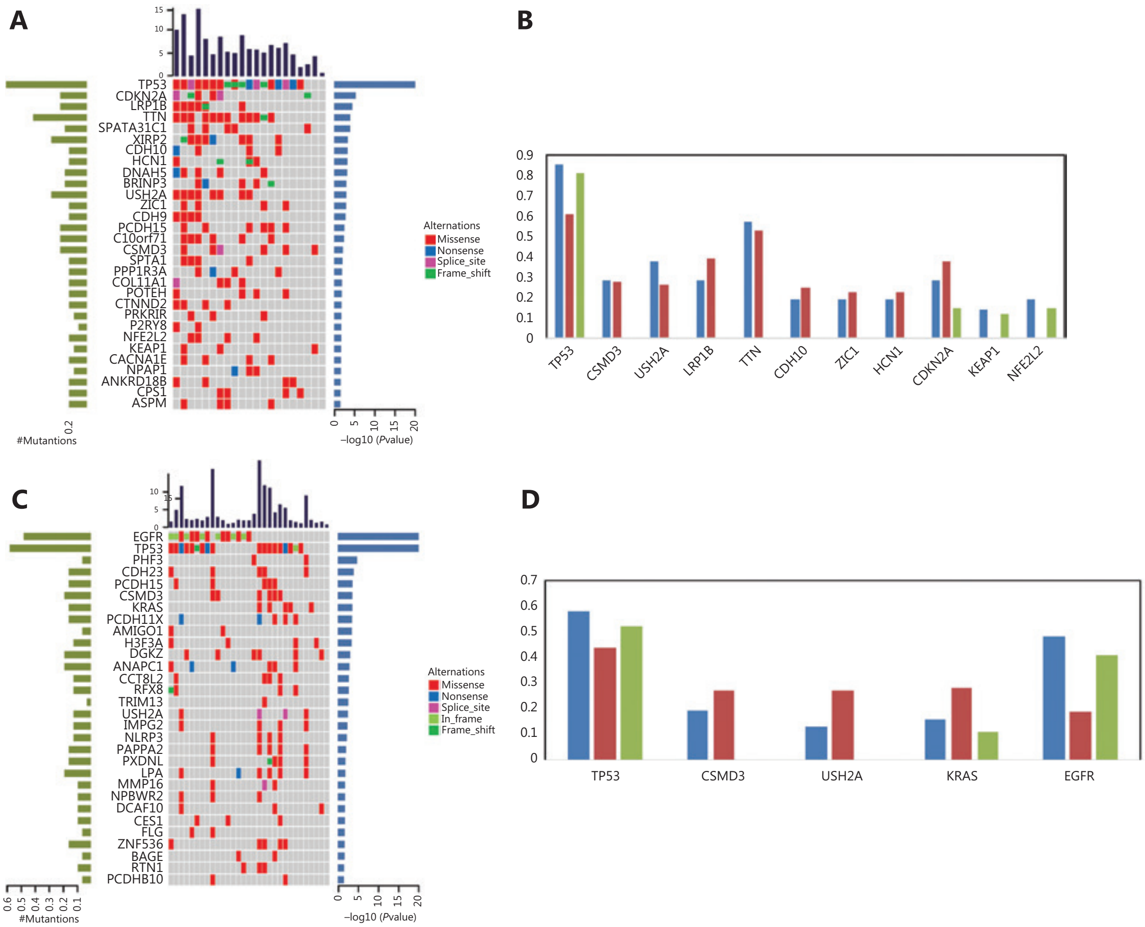

Figure S8 Recurrent mutations in the cohort. A. Genome mutation signature in each patient with lung SCC. B. Recurrent mutations of lung SCC in the current cohort (blue bar), TCGA database (red bar), and reference (green bar). C. Genome mutation signature in each patient with lung Aden. D. Recurrent mutations of lung Aden in the current cohort (blue bar), TCGA database (red bar), and reference (green bar).

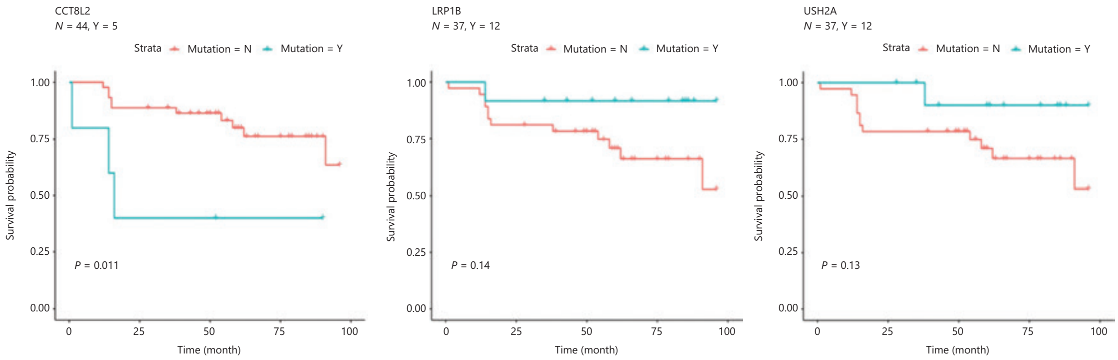

Figure S9 Association between genes with recurrent mutation and DFS after CIK therapy.
